# Supplementary material for: Genetic background and transient prenatal disruption of vitamin A signaling determine susceptibility to airway hyperresponsiveness in mice
Source: bioRxiv. 2025 Nov 3:2025.10.31.685835. Preprint. [Version 1] doi: 10.1101/2025.10.31.685835 (PMC12637646; doi:10.1101/2025.10.31.685835)
Supplement: Supplement 1 — Supplemental Figure 1. Differential effects of prenatal disruption of RA signaling by maternal BMS-containing diet in AJ and B6 mice. (A) Diagram experimental design: maternal administration of control (corn oil) or BMS-containing diet from gestation day 9.5–14.5 to AJ and B6 adult mice. (B) Effect of maternal administration of a diet containing different BMS concentrations (3.75–15 μg/body weight per day (μg/bw/day) on the gross morphology of E14.5 embryos and lungs in both strains. Body truncation and lung hypoplasia at the highest maternal BMS concentration most prominent in AJ embryos compared to B6. These effects were not seen at 3.75 μg/bw/day in either strain. Both controls (corn oil) and BMS (3.75 μg/bw/day) pups reached adulthood. (C) Expression of RA pathway components (Rarb, Cyp26b1, Lrat, Stra6) and Epcam in lung homogenates from E14.5 AJ and B6 embryos (qPCR). Graphs: No downregulation in Epcam expression between control and BMS-exposed lungs in spite of significant Rarb, Cyp26b1, Lrat, Stra6 downregulation by BMS in both strains. Cyp26b1, Lrat and Stra6 expression significantly higher in control AJ compared to control B6, while Rarb is higher in control B6. *p<0.05. **p<0.01. (D) Bulk RNA-seq analysis (Lrat, Stra6, Dhrs3): whole E14.5 lungs from AJ and B6 embryos exposed to maternal BMS (3.75ug/g/bw) diet: downregulation by BMS in both strains and confirming the increased Stra6 and Lrat expression in control AJ compared to B6 control. (E) Effects of prenatal BMS exposure (3.75ug/g/bw) in adult AJ and B6 offsprings: body weight, lung and liver weight relative to body weight. Graphs are mean ± SE, *p<0.05. AJ Control (n=6), AJ BMS (n=6), B6 Control (n=9), B6 BMS (n=6). Supplemental Figure 2. Differential expression of RA-responsive genes in embryonic lungs from AJ and B6 mice underlies the basis for the functional differences between these strains. (A) Schematic overview: workflow for identification of RA–responsive open chromatin regions (RARE-OCRs) enr [file media-1.pdf]

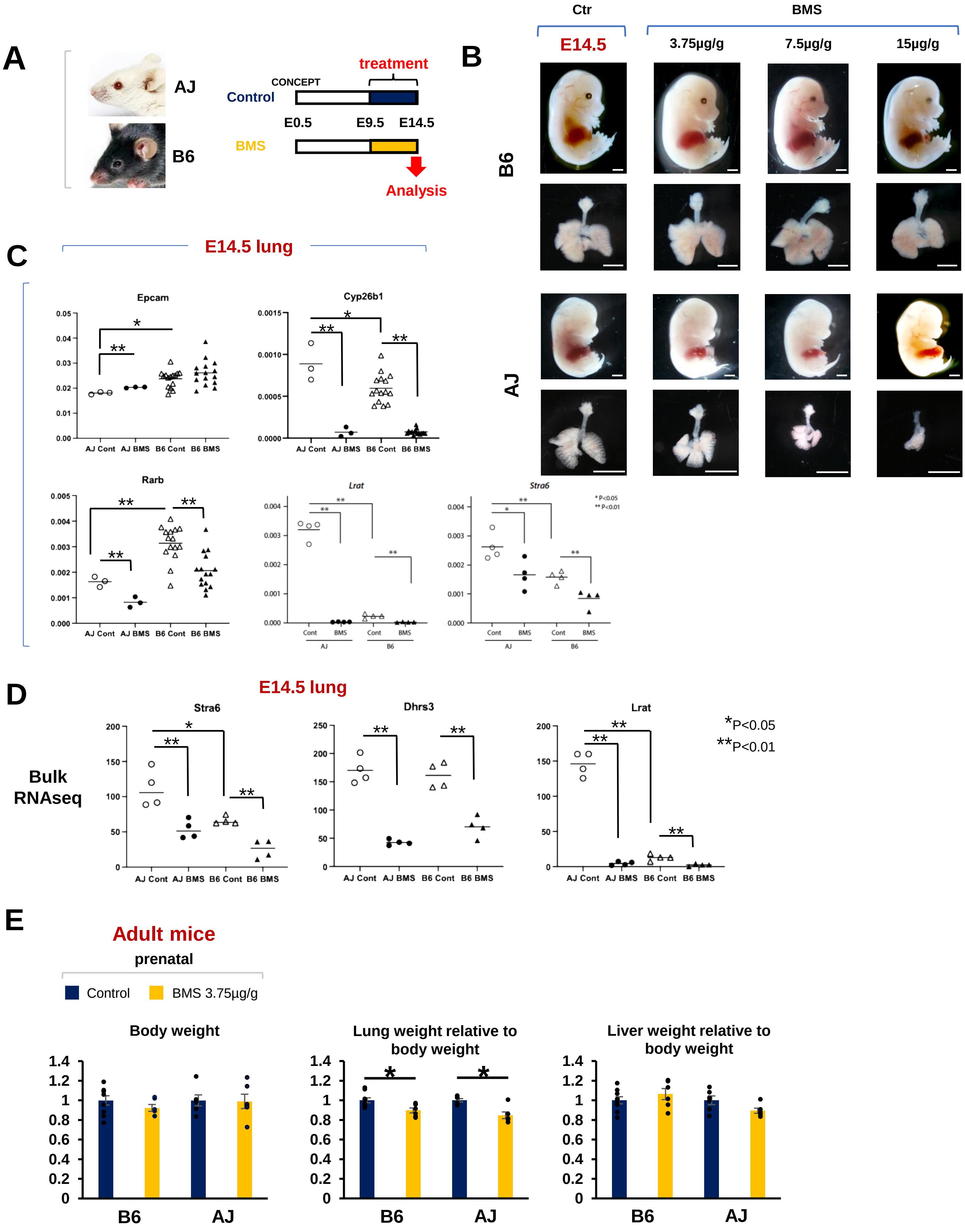

Suppl. Figure 1
Otoshi et al.

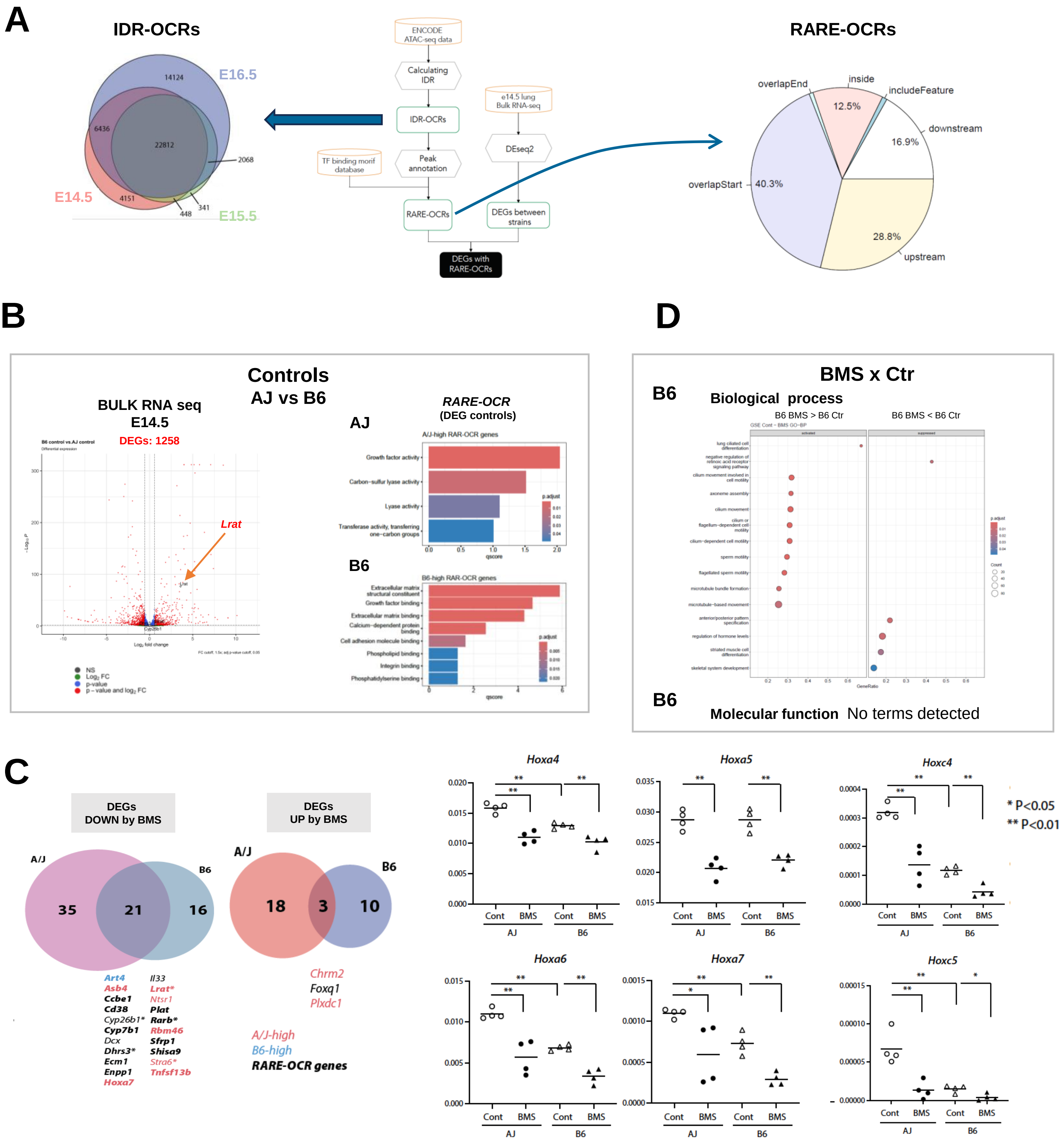

Suppl. Figure 2
Otoshi et al.

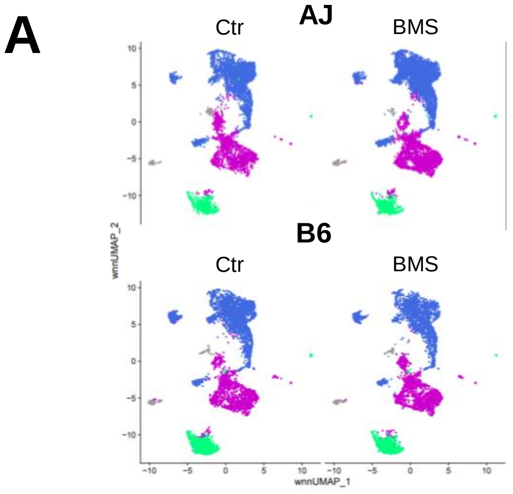

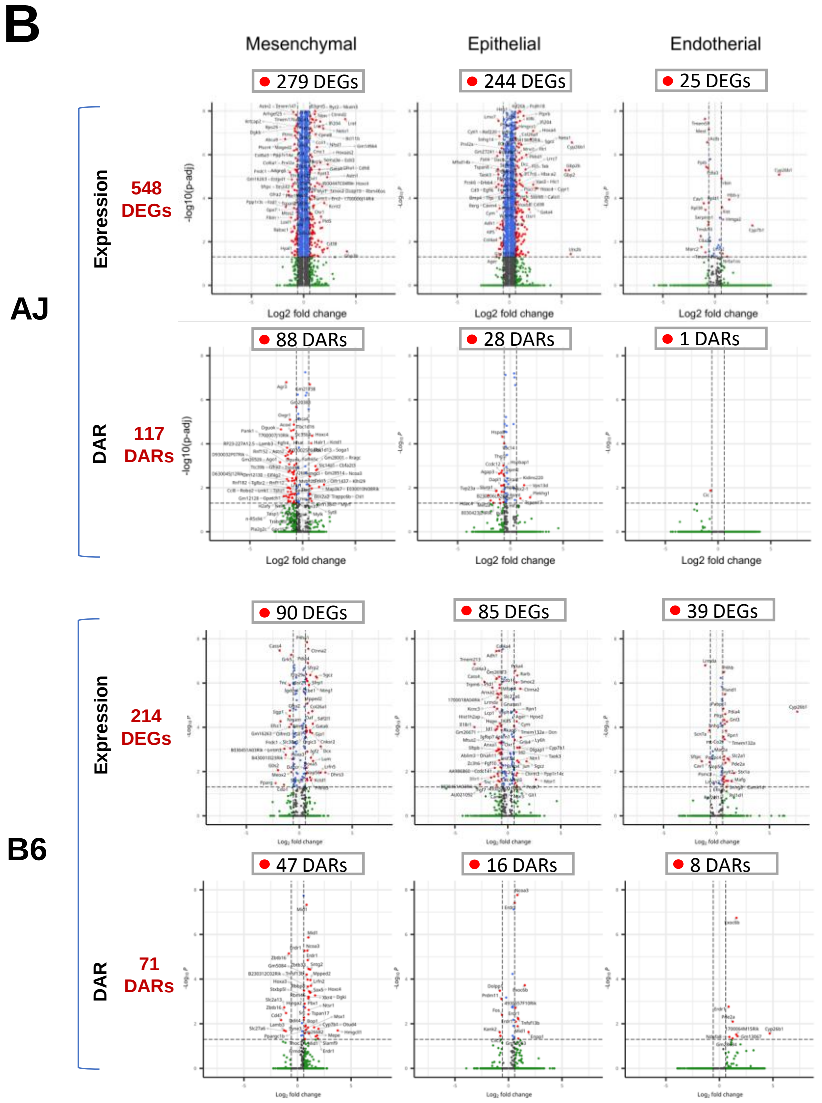

Suppl. Figure 3
Otoshi et al.

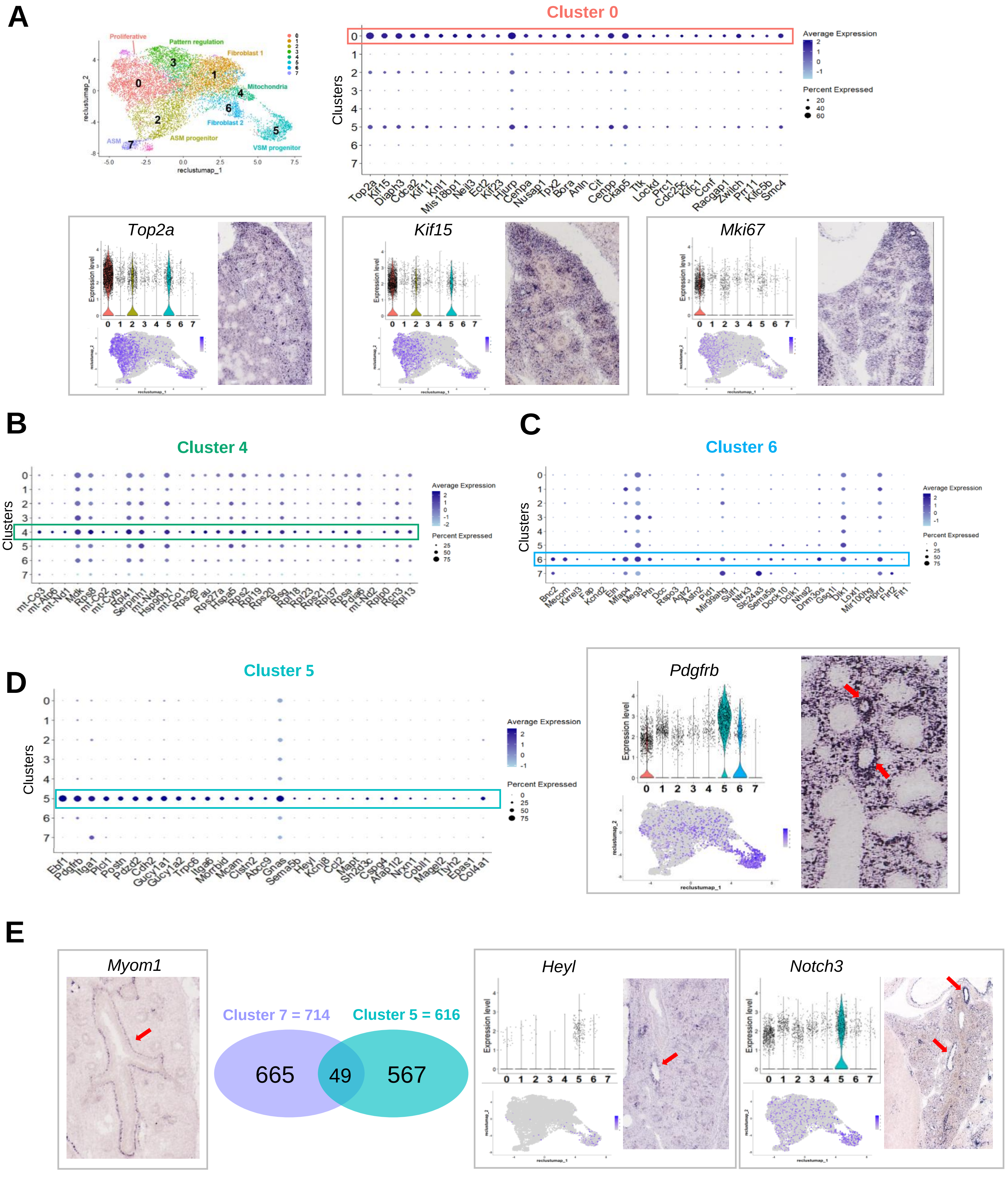

Suppl. Figure 4
Otoshi et al.

Cluster 3

A

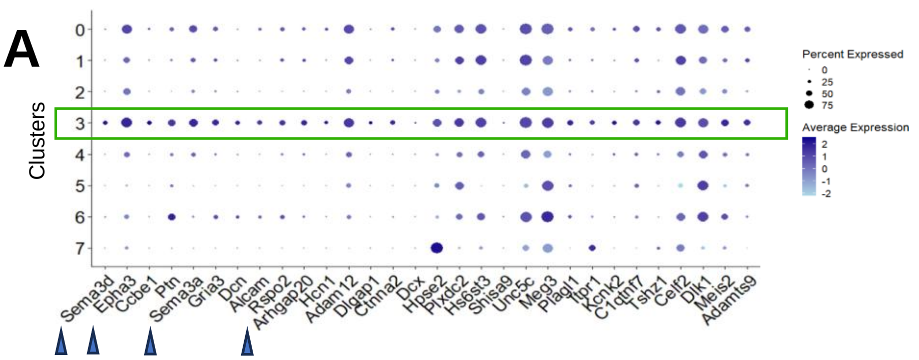

Descartes Cell Types and Tissue

Name	Adjusted p value
Visceral neurons in Lung	0.000006813
ENS neurons in Stomach	0.0006165
Stromal cells in Thymus	0.0008421
Stromal cells in Kidney	0.001193
Stromal cells in Heart	0.009271
Stromal cells in Adrenal	0.009271
Stellate cells in Liver	0.009271
Stromal cells in Lung	0.01401
Vascular endothelial cells in Thymus	0.02486
Stromal cells in Muscle	0.03563

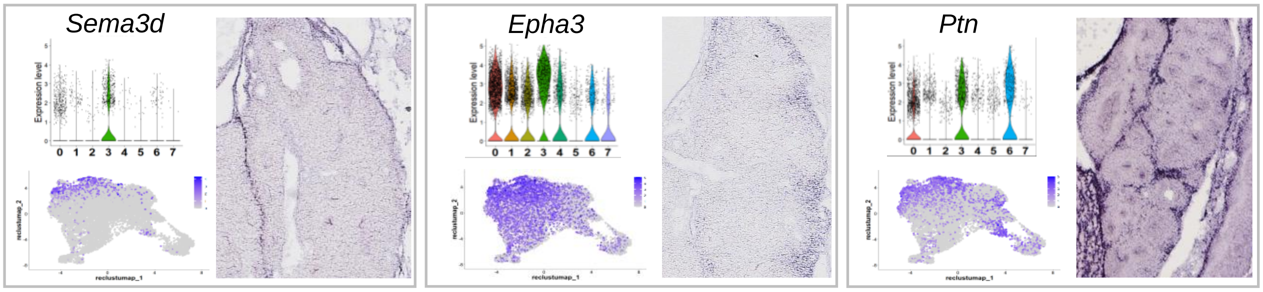

B

Gene	AvgLog2FC	Adj p value ↑
Rspo2	1.52	6.6×10^{-75}
Arhgap20	1.61	6.9×10^{-75}
Hcn1	2.17	1.1×10^{-74}
Adam12	0.94	1.7×10^{-74}
Dlgap1	2.54	1.2×10^{-67}
Ctnna2	1.78	4.4×10^{-65}
Dcx	3.28	1.2×10^{-64}
Hpse2	0.97	9.1×10^{-64}
Plxdc2	0.91	8.6×10^{-59}
Hs6st3	0.77	4.5×10^{-57}
Shisa9	2.61	2.7×10^{-56}
Unc5c	0.53	4.2×10^{-56}
Meg3	0.70	1.6×10^{-51}
Plagl1	1.36	3.1×10^{-49}
Itpr1	1.38	5.5×10^{-49}
Kcnk2	1.66	5.4×10^{-47}
C1qtnf7	1.05	9.2×10^{-47}
Tshz1	1.39	1.3×10^{-45}
Celf2	0.61	2.9×10^{-45}
Dlk1	0.63	8.1×10^{-44}
Meis2	1.01	5.1×10^{-43}
Adamts9	1.07	8.1×10^{-43}
Itga8	0.98	1.8×10^{-41}
Slit3	0.69	1.5×10^{-40}
Fgf10	1.17	2.6×10^{-39}
Capn6	1.86	4.0×10^{-39}
Rian	0.87	4.1×10^{-38}
Wnt2	1.21	2.3×10^{-35}

GO enrichment Cluster 3

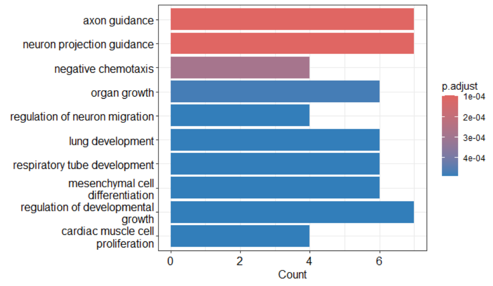

GO term	Genes
axon guidance	*Sema3d, Epha3, Sema3a, Alcam, Unc5c, Slit3, Epha7*
lung development	*Ccbe1, Rspo2, Meg3, Fgf10, Wnt2, Gata6*
mesenchymal cell differentiation	*Sema3d, Epha3, Sema3a, Fgf10, Rian, Wnt2*

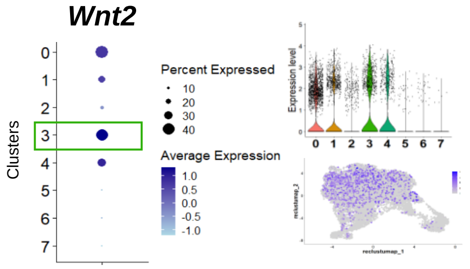

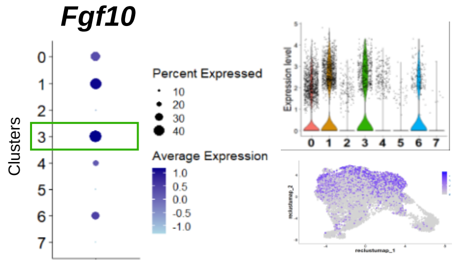

C

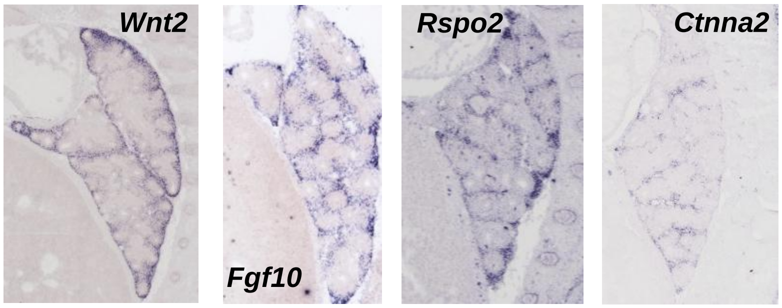

D

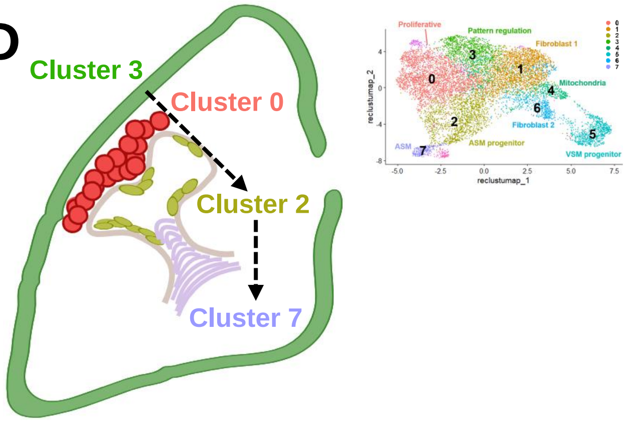

Suppl. Figure 5
Otoshi et al.

A

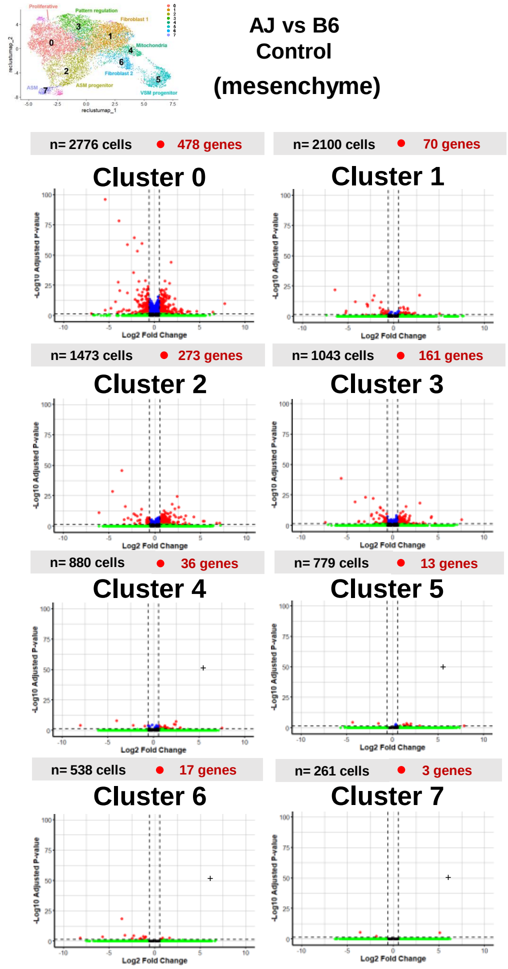

B

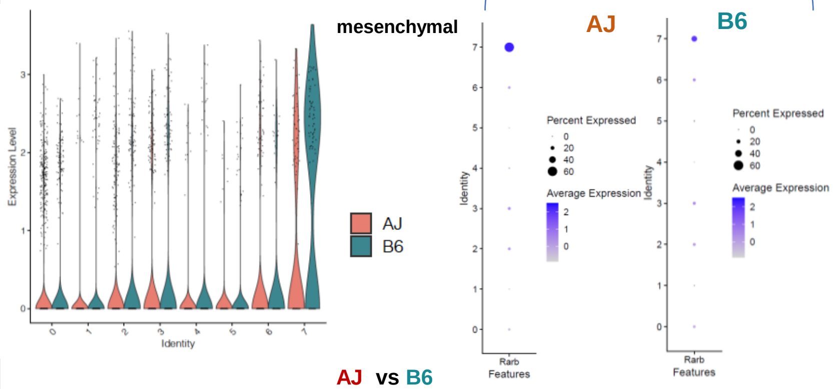

AJ vs B6
Control

Cluster 0

	avg_log2FC	pct.1	pct.2	p_val_adj
Rarb	0.37084952	0.209	0.132	0.259067

Cluster 1

	avg_log2FC	pct.1	pct.2	p_val_adj
Rarb	-0.343422	0.035	0.055	1

Cluster 2

	avg_log2FC	pct.1	pct.2	p_val_adj
Rarb	-0.4443896	0.217	0.176	1

Cluster 3

	avg_log2FC	pct.1	pct.2	p_val_adj
Rarb	0.1572566	0.239	0.169	1

Cluster 4

	avg_log2FC	pct.1	pct.2	p_val_adj
Rarb	-0.39676	0.101	0.068	1

Cluster 5

	avg_log2FC	pct.1	pct.2	p_val_adj
Rarb	0.6644587	0.085	0.05	1

Cluster 6

	avg_log2FC	pct.1	pct.2	p_val_adj
Rarb	0.8317866	0.25	0.149	1

Cluster 7

	avg_log2FC	pct.1	pct.2	p_val_adj
Rarb	-1.137191	0.349	0.648	1

C

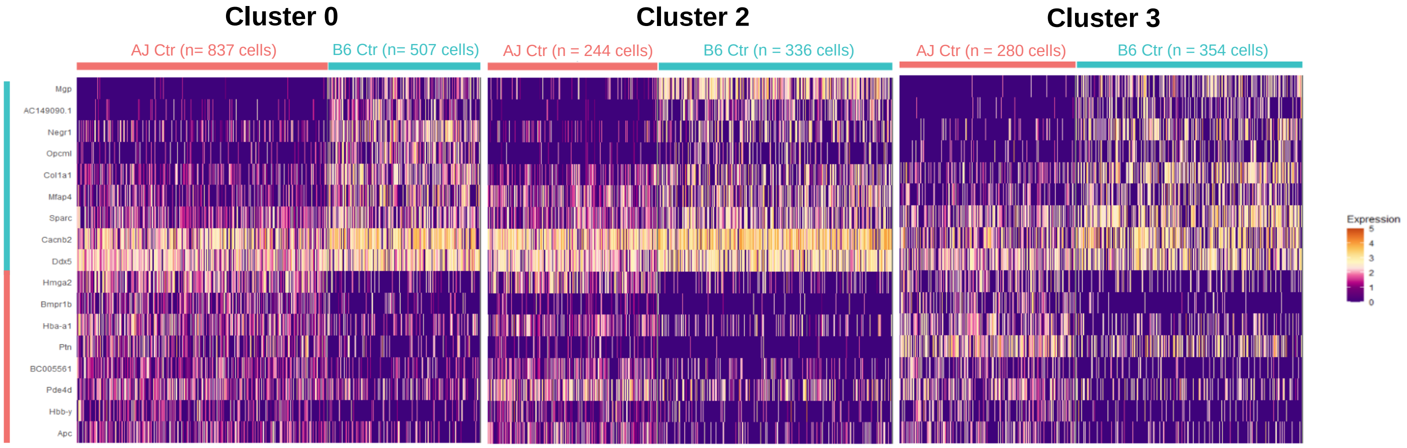

D

Cluster 0

Gene	AvgLog 2FC	Adj p value ↑
Mgp	-5.44	7.8×10^{-97}
AC149090.1	-3.92	2.9×10^{-79}
Negr1	-2.20	3.0×10^{-65}
Gm47283	-1.36	1.8×10^{-60}
Opcml	-2.96	1.7×10^{-59}
Col1a1	-1.90	3.7×10^{-54}
Hmga2	1.85	8.2×10^{-45}
Mid1	-2.30	2.4×10^{-36}
Colec12	-1.01	1.7×10^{-29}
Gm49708	1.18	2.8×10^{-29}
Mfap4	-1.70	4.1×10^{-29}
Myof	-3.98	2.4×10^{-28}
Bmpr1b	1.66	2.1×10^{-27}
Nisch	-0.70	7.9×10^{-25}
Ptprd	-0.69	3.6×10^{-23}
Hba-a1	1.29	8.8×10^{-23}
Slit2	-1.14	1.5×10^{-22}
Hjurp	-0.76	1.9×10^{-22}
Sparc	-0.82	2.9×10^{-22}
Elmo1	-1.89	4.6×10^{-22}
Gpc6	0.82	5.5×10^{-22}

Cluster 2

Gene	AvgLog 2FC	Adj p value ↑
Mgp	-3.59	1.9×10^{-48}
AC149090.1	-4.64	2.0×10^{-29}
Mpped2	2.41	3.1×10^{-25}
Hmga2	1.88	5.2×10^{-19}
Egfem1	-3.21	8.1×10^{-17}
Tmem158	2.60	3.2×10^{-16}
Sdk1	2.19	4.4×10^{-15}
Gm47283	-1.44	8.2×10^{-15}
Mir99ahg	1.00	1.5×10^{-13}
Mtcl1	1.16	6.4×10^{-12}
Kif26b	1.70	6.7×10^{-12}
Myof	-6.11	7.2×10^{-12}
Gm15261	1.32	3.2×10^{-11}
Kcnn2	1.48	6.6×10^{-11}
Rims1	-2.31	7.2×10^{-11}
Magi1	0.73	1.7×10^{-10}
Apc	1.02	1.8×10^{-10}
Prickle1	0.86	2.4×10^{-10}
Col1a1	-1.55	2.5×10^{-10}
BC005561	1.02	4.0×10^{-10}
Negr1	-2.15	5.7×10^{-10}

Cluster 3

Gene	AvgLog 2FC	Adj p value ↑
Mgp	-5.63	1.9×10^{-39}
Negr1	-2.98	7.6×10^{-24}
Col1a1	-2.13	6.2×10^{-23}
AC149090.1	-4.12	3.4×10^{-20}
Bmpr1b	3.00	4.1×10^{-19}
Gm49708	1.48	1.9×10^{-16}
Gm47283	-1.39	1.3×10^{-15}
Col3a1	-1.34	4.3×10^{-15}
Sparc	-1.05	3.4×10^{-13}
Gas5	1.11	3.3×10^{-12}
Dcn	-1.76	4.0×10^{-11}
Hba-a1	0.87	8.0×10^{-11}
Nisch	-0.67	3.6×10^{-10}
Opcml	-2.29	5.0×10^{-10}
Igfbp5	1.42	1.3×10^{-9}
Mfap4	-1.39	3.3×10^{-9}
Zfp292	0.39	6.3×10^{-9}
Foxp1	1.86	1.1×10^{-8}
Mid1	-2.32	1.3×10^{-8}
Col1a2	-1.19	1.4×10^{-8}
Tenm4	0.72	1.4×10^{-8}

E

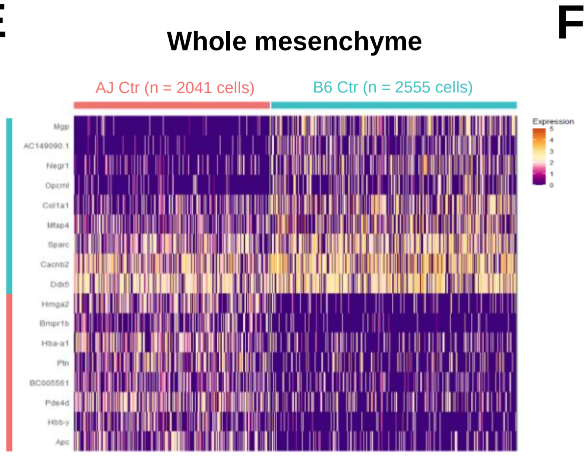

F

**AJ Ctr vs B6 Ctr
Bulk RNA seq**

Gene	AvgLog2FC	Adj p value ↑
Mgp	-2.99	4.0×10^{-251}
Opcml	-2.70	2.0×10^{-96}
Negr1	-2.04	3.0×10^{-72}
Ptn	0.80	3.8×10^{-32}
Mfap4	-0.73	5.9×10^{-19}
Col1a1	-0.75	1.0×10^{-15}
Hmga2	1.10	5.9×10^{-13}
Hbb-y	1.02	2.4×10^{-8}
Hba-a1	0.64	3.2×10^{-2}

**Suppl. Figure 6
Otoshi et al.**

A

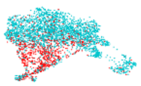

Tgfbi+ cells
(BMS vs Ctrl)
AJ

Top 50 (TOTAL = 337)

Gene	AvgLog2FC	Adj p value ↑
Ddx5	0.61	$3.8{\times}10^{-17}$
Hmga2	-0.98	$1.6{\times}10^{-15}$
Cd63	0.91	$3.2{\times}10^{-15}$
Hbb-y	-2.51	$1.1{\times}10^{-13}$
Epha7	-0.87	$1.3{\times}10^{-12}$
Unc5c	-0.59	$2.5{\times}10^{-11}$
Nr6a1os	-0.64	$2.7{\times}10^{-11}$
Sema3a	-0.82	$5.0{\times}10^{-11}$
Thsd4	-0.41	$1.9{\times}10^{-10}$
Hecw2	-1.24	$2.3{\times}10^{-10}$
Ccbe1	-2.54	$2.9{\times}10^{-10}$
Pdgfra	0.94	$4.7{\times}10^{-10}$
Meg3	-0.39	$7.4{\times}10^{-8}$
2610307P16Rik	-0.55	$9.1{\times}10^{-8}$
Spred1	-0.24	$1.3{\times}10^{-7}$
Prpf40a	-0.55	$1.4{\times}10^{-7}$
Hsp90b1	0.43	$1.7{\times}10^{-7}$
Prex2	-0.83	$2.7{\times}10^{-7}$
Tnc	1.67	$2.7{\times}10^{-7}$
Bmpr1b	-0.81	$4.0{\times}10^{-7}$
Pdzd2	-0.67	$5.0{\times}10^{-7}$
Pdia3	0.34	$6.8{\times}10^{-7}$
A630089N07Rik	-0.24	$8.5{\times}10^{-7}$
Ppic	0.91	$9.8{\times}10^{-7}$
Ptk2	-0.42	$1.0{\times}10^{-6}$
Plagl1	-0.69	$1.1{\times}10^{-6}$
Lrfn5	-3.29	$1.9{\times}10^{-6}$
Itm2b	0.88	$2.9{\times}10^{-6}$
Trim24	-0.40	$3.2{\times}10^{-6}$
Dnmt3a	0.29	$3.9{\times}10^{-6}$
Tshz3	-0.43	$5.8{\times}10^{-6}$
Ccny	-0.29	$5.9{\times}10^{-6}$
P3h2	-1.23	$7.3{\times}10^{-6}$
Mfap4	0.65	$7.5{\times}10^{-6}$
Tent2	-0.44	$9.8{\times}10^{-6}$
Nisch	0.40	$1.0{\times}10^{-5}$
Cyp7b1	-1.09	$1.2{\times}10^{-5}$
Slit3	-0.51	$1.3{\times}10^{-5}$
Hotairm1	-1.21	$1.3{\times}10^{-5}$
Hba-a1	-1.40	$1.5{\times}10^{-5}$
Laptm4a	0.39	$1.7{\times}10^{-5}$
Sulf1	-1.06	$1.9{\times}10^{-5}$
Rbm46	-0.87	$2.0{\times}10^{-5}$
Dlc1	-0.29	$2.1{\times}10^{-5}$
Slc25a36	-0.26	$3.0{\times}10^{-5}$
Tnrc6a	-0.19	$3.0{\times}10^{-5}$
Frmd4a	-0.25	$3.0{\times}10^{-5}$
Ptbp2	-0.46	$3.1{\times}10^{-5}$
Cd81	0.39	$3.5{\times}10^{-5}$
Rspo2	-0.79	$3.5{\times}10^{-5}$

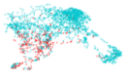

Tgfbi+ cells
(BMS vs Ctrl)
B6

TOTAL = 6

Gene	AvgLog2FC	Adj p value ↑
Serpinh1	-0.79	$8.2{\times}10^{-3}$
Hspa5	-0.80	$1.2{\times}10^{-2}$
Mdk	-0.60	$1.3{\times}10^{-2}$
Ptprk	0.57	$1.6{\times}10^{-2}$
1700018A04Rik	1.00	$3.1{\times}10^{-2}$
Lcorl	0.48	$3.7{\times}10^{-2}$

B

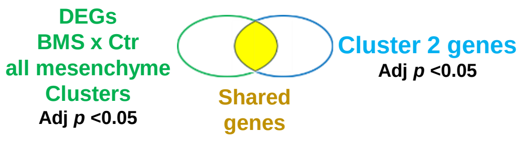

CLUSTER 2 GENES DOWNREGULATED BY BMS

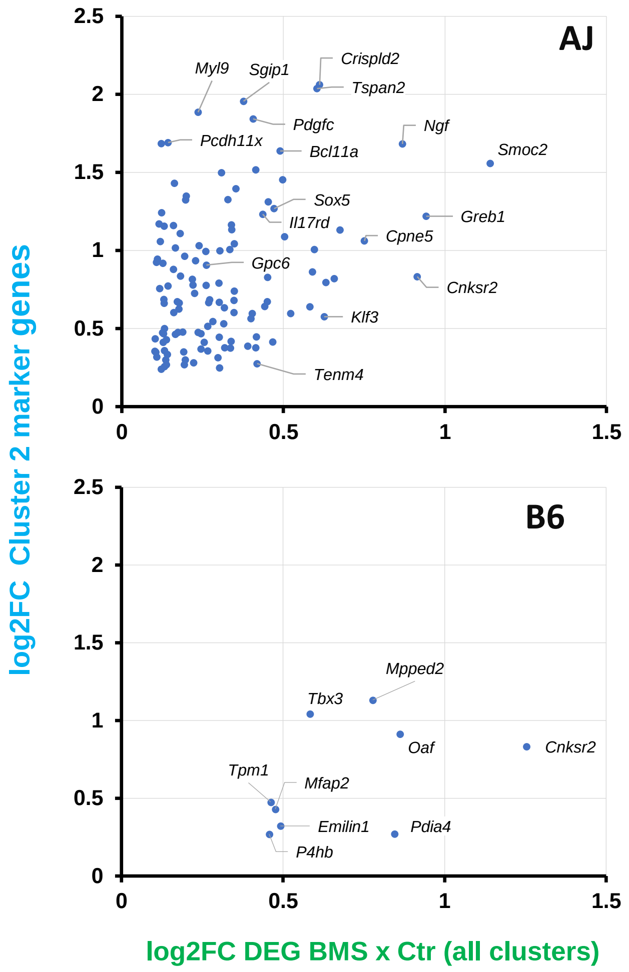

C

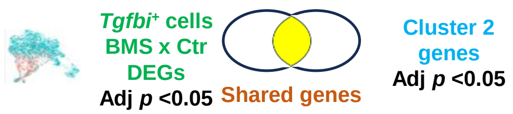

CLUSTER 2 GENES UPREGULATED BY BMS IN *Tgfbi+* CELLS

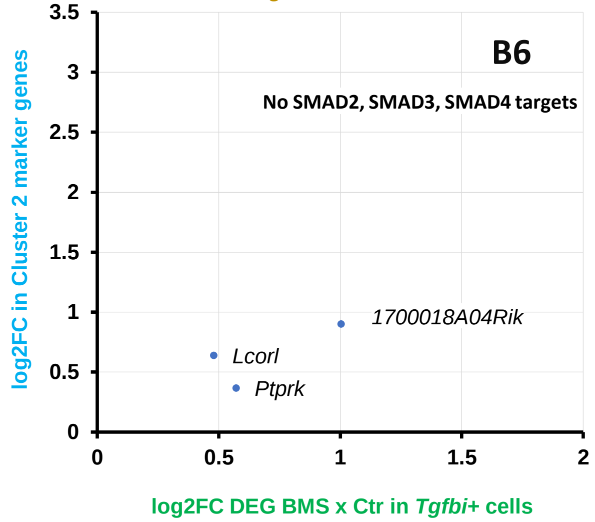

Suppl. Figure 7
Otoshi et al.
